# Prediction of herbal compatibility for colorectal adenoma treatment based on graph neural networks

**DOI:** 10.1186/s13020-025-01082-5

**Published:** 2025-03-05

**Authors:** Limei Gu, Yinuo Ma, Shunji Liu, Qinchang Zhang, Qiang Zhang, Ping Ma, Dongfang Huang, Haibo Cheng, Yang Sun, Tingsheng Ling

**Affiliations:** 1 Gastrointestinal Endoscopy Center, Affiliated Hospital of Nanjing University of Chinese Medicine, Jiangsu Provincehospital of Chinese Medicine, Nanjing, 210029 China; 2 State Key Laboratory of Pharmaceutical Biotechnology, School of Life Sciences, Chemistry and Biomedicine Innovation Center (Chembic), Nanjing University, 163 Xianlin Avenue, Nanjing, 210023 China; 3 Jiangsu Collaborative Innovation Center of Traditional Chinese Medicine in Prevention and Treatment of Tumor, The First Clinical Medical College, Nanjing University of Chinese Medicine, 138 Xianlin Avenue, Nanjing, 210023 Jiangsu China

**Keywords:** Colorectal adenoma, Traditional Chinese medicine, Graph neural network

## Abstract

Colorectal adenoma is a common precancerous lesion with a high risk of malignant transformation. Traditional Chinese medicine and its complex prescriptions have shown promising efficacy in the treatment of adenomas; however, there remains a lack of systematic understanding regarding the compatibility patterns within these prescriptions, as well as an effective model for predicting therapeutic outcomes. In this study, we collected numerous TCM prescriptions and their components, recommended by experts for the treatment of colorectal adenoma, and developed a heterogeneous graph neural network model to predict the compatibility strength and probability among the herbs within these prescriptions. This model delineates the complex relationships among herbs, active compounds, and molecular targets, allowing for a quantification of the interactions and compatibility potential among the herbs. Using this model, we identified high-potential therapeutic prescriptions from clinical prescription records and identified their active components through network pharmacology. Through this approach, we aim to provide a theoretical foundation for the clinical TCM treatment of colorectal adenoma, foster the discovery of new prescriptions to optimize the therapeutic efficacy of TCM, and ultimately advance the field of cancer prevention and treatment based on traditional Chinese medicine.

## Introduction

Traditional Chinese Medicine (TCM), a hallmark of Chinese culture, boasts a rich history and extensive clinical experience. With its emphasis on holistic health and the principles of syndrome differentiation, TCM has demonstrated significant advantages in managing chronic diseases [1]. Colorectal adenoma is a common precancerous condition with a high risk of progressing to colorectal cancer. It poses a critical challenge for healthcare systems worldwide [2]. Its effective management is essential for reducing colorectal cancer incidence.

In recent years, research on the potential and mechanisms of TCM in the treatment of colorectal adenomas has gradually increased [3]. To more effectively utilize Chinese herbal medicine, TCM prescriptions are commonly employed in clinical practice to address the complex conditions of patients. Clinical studies have demonstrated that these prescriptions exhibit significant efficacy in reducing adenoma recurrence and improving patients' quality of life [4, 5]. However, the complexity of TCM prescriptions, which involve the synergistic effects of multiple herbs and their components, presents a challenge in understanding their pharmacological mechanisms and therapeutic outcomes [6]. Existing studies indicate that TCM prescriptions treat colorectal adenomas through multiple pathways, particularly in regulating the gut microbiota [7, 8], metabolites [9], and immune responses [9, 10]. Due to the complexity of pharmacological actions and the interactions and compatibility relationships between herbs, the development of new TCM prescriptions not only faces substantial challenges but also relies heavily on the guidance of clinical experts.

In the mining and recommendation of TCM prescriptions, existing studies predominantly rely on single data analysis methods, such as association rule mining or regression analysis, which struggle to capture the nonlinear relationships and intricate network characteristics of herbal compatibility [11–13]. The advent of graph neural networks (GNNs) presents an innovative approach to untangle the complexities of TCM prescriptions. GNNs have gained prominence for their ability to model relational data and uncover intricate patterns in networks [14, 15]. In recent years, significant advancements have been made in the intersection of TCM and GNNs. For instance, research by Zhou et al. and Zhao et al. utilizes expert knowledge and herbal characteristics to construct models for prescription recommendation. Their work not only enhances the accuracy of TCM prescription recommendations but also provides data support for personalized treatment [16, 17]. Moreover, Han et al. applied GNN models to effectively predict new indications for existing prescriptions [18]. These studies highlight the potential of GNNs in handling the intricate data of TCM and lay the groundwork for innovative applications in the field. The success of such interdisciplinary research demonstrates how modern computational techniques can be effectively integrated with traditional medical knowledge to advance precision medicine and improve patient treatment outcomes.

Existing GNN models have achieved certain successes in TCM formula recommendation and indication prediction, but they mainly focus on the treatment of general diseases and have not fully addressed the balance between the complex theories of herbal compatibility and the demands for individualized treatment. These models typically rely on the general properties of herbs, neglecting disease specificity and individual differences, which limits the treatment effectiveness for specific diseases. This study seeks to model the compatibility patterns within TCM prescriptions for colorectal adenoma by GNN. Through this approach, we aim to quantify the interactions and potential synergies among various herbs and their components. By integrating modern computational techniques with traditional medical knowledge, this research provides new insights into optimizing TCM prescriptions. The application of GNNs in this domain not only elucidates the underlying compatibility dynamics of TCM prescriptions but also aids in the discovery of novel therapeutic combinations, promoting the effective use of TCM in colorectal cancer prevention and treatment.

The innovation of this study lies in its focus on specific diseases such as colorectal adenoma. Based on expert knowledge, it leverages GNN to explore the potential compatibility patterns among herbs, accurately capturing their interactions and synergistic effects to optimize treatment plans. Compared to existing models, this research not only improves the accuracy of formula recommendations but also better meets the needs of personalized treatment, offering a new breakthrough for precision medicine.

## Materials and methods

### Graph structure

In this study, we gathered data on 72 TCM prescriptions used to treat colorectal adenoma from clinical trials and published literature. Each prescription comprises multiple herbs, each of which may contain various active small-molecule compounds and their corresponding targets. By integrating this information, we constructed a heterogeneous graph network to comprehensively represent the complex relationships among herbs, small molecules, and targets.

This heterogeneous network includes three types of nodes: herbs, small molecules, and targets. The network contains 122 herb nodes, 234 small-molecule nodes, and 657 target nodes, interconnected by 24,608 edges (Table 1).

**Table 1:** Composition of the heterogeneous diagram

Type	Quantity	Features
Herb	122	Node embeddings
Small Molecule	234	Node embeddings, MACCS fingerprints
Target	657	Node embeddings, GO (Gene Ontology) terms
Herb-Herb	2962	–
Herb-Molecule	1436	–
Molecule-Target	20,426	–

### Model structure

**Fig. 1:**
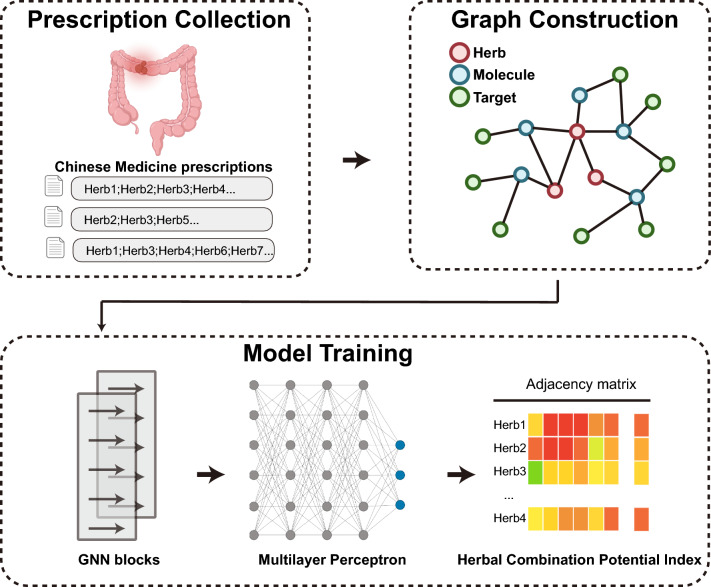
Model workflow for herbal compatibility prediction

To quantify the potential compatibility of TCM prescriptions, we have developed a model framework based on GNN. The GNN model consists of two components: (1) The Graph Convolutional Network (GCN) layers propagate and aggregate features within the heterogeneous graph structure, capturing both local network topological information and the characteristics of each herb within the formulation. The first GCN layer extracts the local neighborhood features of each node and embeds them into a lower-dimensional feature space; subsequently, the second GCN layer extracts higher-order neighborhood features, providing a more comprehensive representation of the herbs within the network; (2) A Multi-Layer Perceptron (MLP) is then employed to predict the compatibility between herbs. This process is framed as a supervised learning task, with supervisory signals derived from the known herb-herb compatibility values calculated from the herbal prescription information we collected (Fig. 1). This design enables the model to effectively integrate structural information with expert knowledge in herb compatibility prediction tasks, thereby enhancing both the predictive accuracy and interpretability of the model.

#### Data collection and filtering

This study conducted a comprehensive computer-based search across several databases, including China National Knowledge Infrastructure (CNKI), Wanfang Data Knowledge Service Platform, VIP Database, and PubMed, with a retrieval time span from the establishment of each database to March 2024. Search terms included "colorectal adenomas," "colorectal polyps," "traditional Chinese medicine," and "herb," among other related keywords.

The inclusion criteria comprised clinical trials based on the clinical experiences of renowned TCM practitioners in the treatment of colorectal adenomas, specifically randomized controlled trials (RCTs) or clinical studies on traditional Chinese medicine. The literature must explicitly list the drug composition of the formulation and evaluate its efficacy. There were no restrictions on the types of TCM formulations used, and decoctions, granules, capsules, and other forms were all eligible for inclusion. If multiple studies employed the same prescription or if the same article was published multiple times, only one study was retained. Studies were excluded if they were duplicates, lacked a clear listing of the prescription composition or efficacy, were review articles, or had incomplete or unclear descriptions of the formulations. These criteria ensured that the included studies provided reliable clinical data and accurate prescription information, thereby offering high-quality reference material for subsequent analysis.

Molecular and target information, as well as their interactions, were sourced from the Traditional Chinese Medicine System Pharmacology Database and Analysis Platform (TCMSP) [19]. Small molecules were selected based on the criteria of oral bioavailability (OB) ≥ 30 and drug likeness (DL) ≥ 0.18. Verified targets were employed to ensure that the selected molecules and their corresponding interactions held reliability and relevance for further research.

#### Herb-herb compatibility calculation

We quantified the compatibility of each pair of herbs using their co-occurrence frequency across 72 prescriptions. The formula is given by:

$$C_{ij} \, = \, (N_{ij} \, / \, \sum\nolimits_{k = 1} m \, (n_{k} (n_{k} \, - { 1}) \, /{ 2})) \, \times { 1}000$$

where *C*_ij_ denotes the compatibility score between herb *i* and herb *j*, *N*_ij_ represents the number of times herbs *i* and *j* co-occur across all prescriptions, and *m* is the total number of prescriptions.

#### Feature aggregation

In the feature aggregation process, we implemented a Graph Convolutional Network (GCN) that updates each node's representation by aggregating features from neighboring nodes. Specifically, we utilized the SAGEConv operator to achieve this aggregation. Each layer of the GCN updates the node's feature vector, incorporating not only the node's own information but also the features from its neighborhood. The GCN layer's update formula is as follows:

$${\text{h}}_{v}^{{\left( {l + 1} \right)}} = {\text{ RELU }}\left( {{\mathbf{W}}^{\left( 1 \right)} \cdot {\text{CONCAT}}\left( {{\text{h}}_{v}^{\left( 1 \right)} \, ,{\text{ h}}_{n} \left( {\text{v}} \right)^{\left( 1 \right)} } \right)} \right)$$

Where h_v_^(l+1)^ represents the embedding of node *v* at layer l+ 1, **W**^(1)^ is the weight matrix for layer l*,* RELU is a non-linear activation function, and CONCAT is the concatenation operation, merging the current features of the node and its neighborhood. By stacking multiple GCN layers, the model can extract more intricate features from local neighborhoods, yielding more semantically meaningful node representations.

#### Herb relationship predictor

In the herb relationship prediction module, we used a Multi-Layer Perceptron (MLP) as the relationship predictor for pairs of herb nodes. For any pair of herb nodes, we first extract their embedding vectors **H**_vi_ and **H**_vj_ and then concatenate these vectors to form a new feature representation **H**_ij_:

$${\mathbf{H}}_{ij} \, = {\text{ CONCAT}}({\mathbf{H}}_{vi} ,{\mathbf{H}}_{vj} )$$

Next, this concatenated feature **H**_ij_ is fed into the MLP network to predict the relationship strength, yielding a final score ŷ_ij_ that represents the relationship strength between the two herb nodes:

$$\widehat{y}_{ij} \, = {\text{ MLP}}({\mathbf{H}}_{ij} )$$

This predictor uses a non-linear mapping to capture the complex relationship structure between herb nodes.

#### Loss calculation

To train the model, we adopted the Mean Squared Error(MSE) loss function to evaluate the deviation between predicted values and true values. The formula for this loss function is:

$${\mathbf{L}} = { 1}/{\mathbf{M}}\sum\nolimits_{i}^{ = 1M} {\left( {y_{i} - \widehat{y}_{i} } \right)}^{2}$$

Where **M** represents the number of herb node pairs, *y*_i_ is the true relationship value for the *i*-th pair of herb nodes, and ŷ_i_ is the predicted value for that pair. The MSE loss function measures the model’s accuracy by computing the squared difference between predicted and true values. During training, by minimizing the MSE loss, the model parameters are updated, leading to progressive convergence and improved accuracy in predicting herb node relationships.

#### Hyperparameter selection and cross-validation

We employed a combination of grid search and five-fold cross-validation to evaluate the model's generalization ability and optimize the hyperparameters. By using MSE and the Coefficient of Determination (R^2^) as evaluation metrics, we systematically compared the model's performance across different hyperparameter combinations (Table 2). The results demonstrated that the combination of 256 hidden layer nodes and a learning rate of 0.001 exhibited superior performance in all experiments. This configuration achieved favorable results across multiple evaluation metrics, indicating strong generalization ability of the model under this parameter setting.

**Table 2:** Hyperparameter search space and model performance

Hidden Channels	Learning Rate (lr)	MSE	R^2^
64	0.0001	0.8503	0.0723
64	0.001	0.1462	0.8262
64	0.01	0.1012	0.8837
128	0.0001	0.7266	0.2010
128	0.001	0.0792	0.9060
128	0.01	0.3829	0.5698
256	0.0001	0.4851	0.4713
256	0.001	0.0772	0.9065
256	0.01	1.2507	/
512	0.0001	0.1591	0.8095
512	0.001	0.0873	0.8965
512	0.01	1.1890	/

### Herbal combination potential index calculation and prescription efficacy prediction

The Herbal Combination Potential Index (HCPI) is used to quantify the interaction potential between two herbs. Specifically, we utilize the trained model to predict the interaction strength between two herb nodes, yielding a predicted score ŷ. To further normalize this value and enhance its interpretability, we apply a log₂ transformation, defining the HCPI as follows:

$${\text{HCPI}}\, = \,\log_{2} (\widehat{y}\, + \,{1}).$$

where ŷ denotes the predicted interaction strength between the two herb nodes as estimated by the model.

For prescription efficacy prediction, we hypothesize that higher compatibility between herbs within a prescription corresponds to greater formulation efficacy, exerting a positive influence on overall therapeutic outcomes. Based on this assumption, we quantify the potential formulation effect by computing the average herb-to-herb compatibility within each prescription. To better align with the requirements of the predictive model, we take the logarithm (log₂) of this average compatibility value, using it as an indicator for efficacy prediction.

### Gene ontology enrichment analysis

In this study, we utilized the functions provided by the ClusterProfiler package to perform Gene Ontology (GO) enrichment analysis [20]. The analysis was conducted using the default method, which involves the hypergeometric distribution test. This statistical approach is implemented to determine the significance of GO term enrichment within a given set of genes. Furthermore, to control the false discovery rate associated with multiple hypothesis testing, we applied the Benjamini & Hochberg method.

### Construction and analysis of PPI network

In this study, we employed the STRING database in conjunction with Cytoscape software to construct a protein–protein interaction (PPI) network for the intersecting targets of the prescription and colorectal adenoma. Initially, we retrieved the relevant protein interaction data from the STRING database, which was then imported into Cytoscape for the construction and visualization of the PPI network. Within Cytoscape, we utilized the Analyzer Network plugin to perform an in-depth analysis of the constructed PPI network.

### Molecular docking simulation

In this study, AutoDock Vina was utilized for molecular docking simulations to investigate the interactions between ligands and the receptor protein. The receptor protein structure was obtained from the Protein Data Bank (PDB) and preprocessed using software to remove water molecules and ligands, followed by the addition of polar hydrogen atoms and assignment of Gasteiger charges. Ligands were either retrieved from databases or generated using chemical drawing software, with subsequent addition of hydrogens and charge computation. Both receptor and ligand files were then converted to the necessary PDBQT format for AutoDock Vina. The docking grid was centered on the active site of the receptor, and docking was performed to evaluate binding affinities.

### CCK-8 cell viability assay

Digestion, centrifugation, and suspension counting of HCT116 and HT29 with moderate density were performed. Cells of 5*10^3^/ well were inoculated on a 96-well plate and cultured at 37℃ and 5% CO^2^ for 24 h. The drug concentration of Oxyberberine was 0, 0.3, 3, 7.5, 15, 30, 75, 150, 300, 750 μM, and the drug concentration of Maackiain was 0, 5, 10, 20, 50, 100, 200 nM. At least three.

wells were added to the 96-well plate at each concentration and cultured at 37℃ and 5%CO_2_ for 24 h. After 24 h of culture, 10μL of CCK-8 solution was added to each well, and cultured at 37℃ and 5%CO_2_ for 2 h. Absorbance was measured at 450 nm by enzyme-linked immunoassay, and the cell viability was calculated.

## Results

### Analysis of herbal applications and component characteristics

**Fig. 2:**
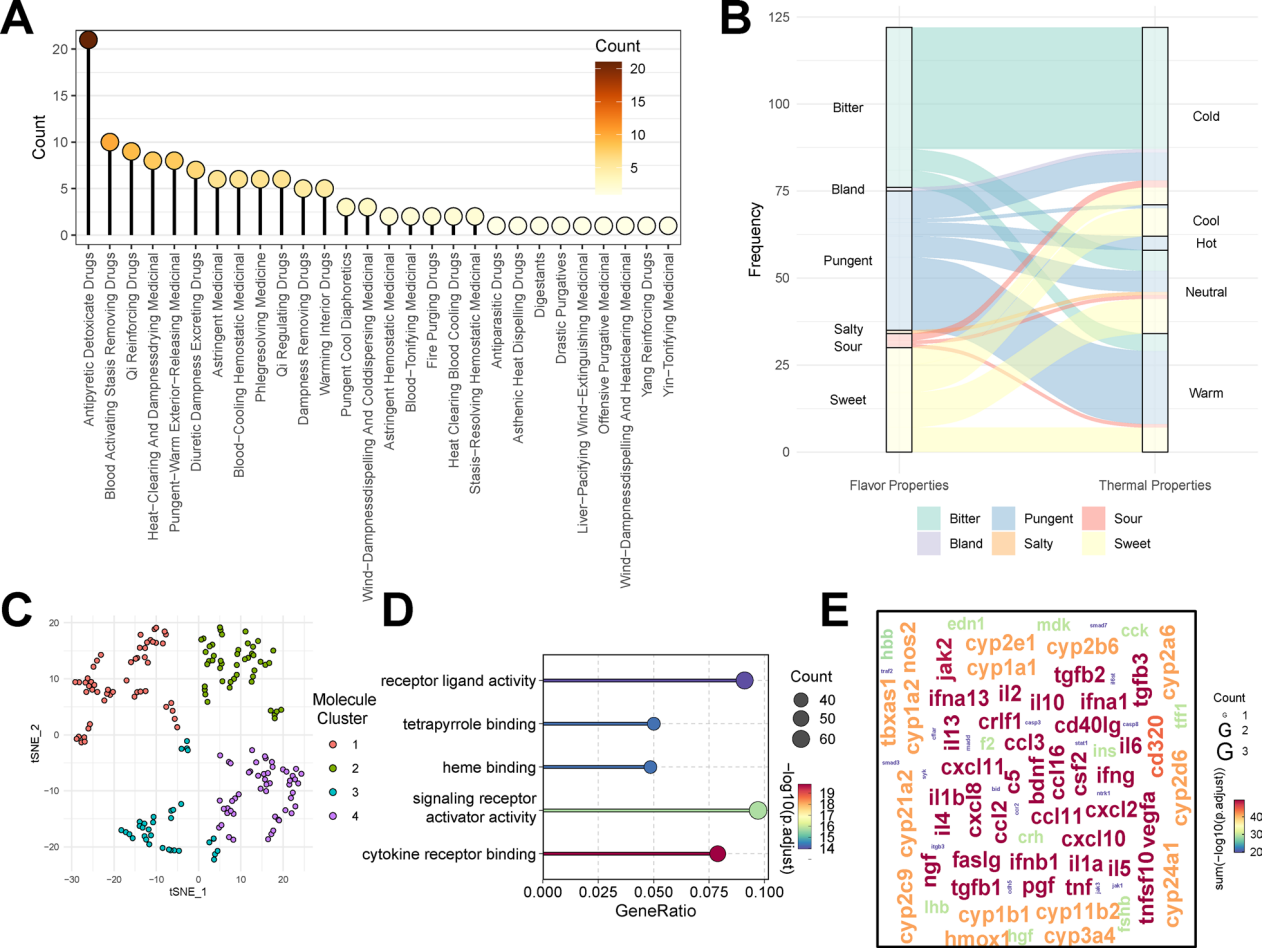
Analysis of herbal categories, properties, molecular composition, and target enrichment in colorectal adenoma treatments. **A** Distribution of Herbal Categories for Colorectal Adenoma Treatment. **B** Distribution of Herbal Properties (Nature and Flavor). **C** t-SNE Clustering of Small Molecule Components Based on MACCS Fingerprints. **D** GO Enrichment Analysis of Herb Targets. **E** Word Cloud of GO Enrichment Analysis Terms

Among the various categories of traditional Chinese medicine (TCM) used for colorectal adenoma treatment, Antipyretic Detoxicate Drugs are the most numerous (Fig. 2A). These herbs possess functions of heat-clearing, detoxifying, anti-inflammatory, antibacterial, and immune-enhancing properties, and they are often employed to relieve pathological conditions caused by internal heat or inflammation. As such, they are widely applied in cancer therapy. An analysis of the properties of these herbs in terms of nature and flavor (Fig. 2B) reveals that most are bitter, a taste linked in TCM theory with functions such as clearing heat, reducing fire, and detoxification. Additionally, bitter herbs are frequently cold in nature, supporting their role in reducing internal heat and inflammation.

Furthermore, we conducted a clustering analysis of small molecular components in these herbs based on MACCS fingerprints and visualized their spatial distribution using the t-SNE algorithm (Fig. 2C). The results indicate that these small molecules form four main clusters, suggesting potential functional classification based on biological roles. Each cluster may correspond to different physiological functions or molecular mechanisms, such as anti-inflammatory, antioxidant, or immune-regulatory effects, providing valuable insights into the therapeutic action of these herbs in colorectal adenoma treatment.

Finally, GO enrichment analysis of small molecule targets indicates significant enrichment in biological processes such as receptor-ligand activity, tetrapyrrole binding, heme binding, and signal receptor activation (Fig. 2D). These functional categories are closely associated with inflammatory responses, cellular signaling, and metabolic regulation, suggesting a multi-target regulatory mechanism by which TCM prescriptions may exert therapeutic effects in cancer treatment. Key targets, such as IFNG, TNF, and CXCL10, play crucial roles in immune response regulation, cell proliferation, and apoptosis. Their activation and regulation may explain the diverse biological effects of TCM prescriptions in the treatment of colorectal adenoma (Fig. 2E).

### Screening of high-potential herbal pairs based on model predictions and network feature analysis

**Fig. 3:**
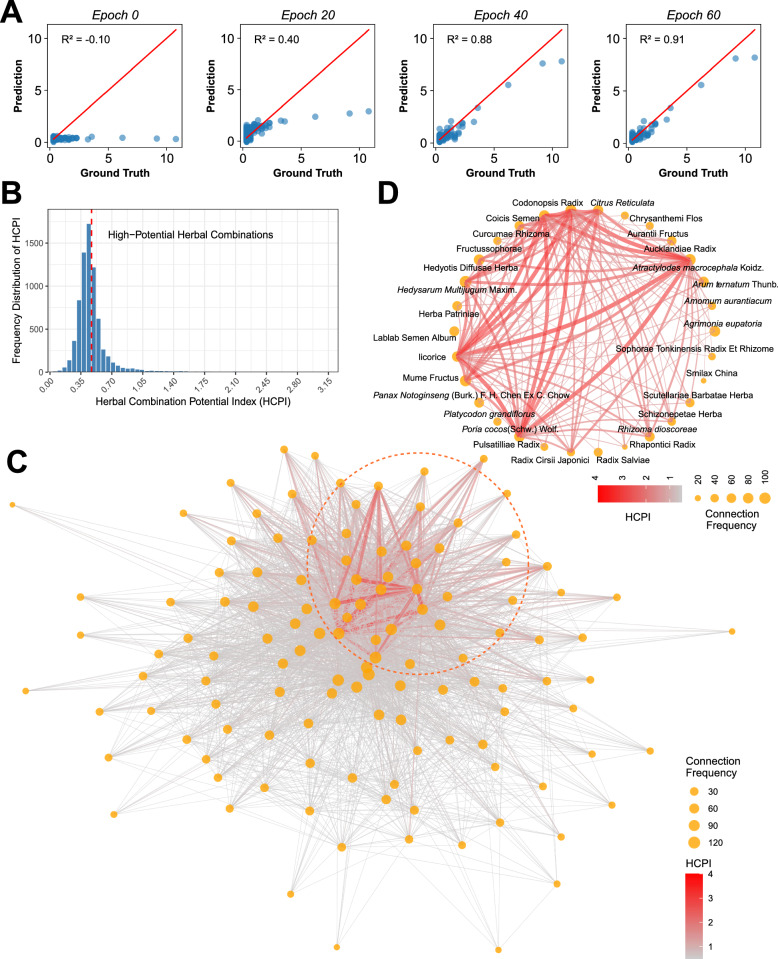
Analysis of herb-herb pairing potential based on model predictions. **A** Comparison of Model Predictions and Actual Values. **B** High-Potential Herb Pair Screening. **C** Herb-Herb Pairing Network Structure. **D** Core Network of High-Frequency, High-Potential Herb Pairs

Through multiple training rounds, we observed a gradual increase in predictive accuracy as the training iterations progressed, with the final model achieving a high fit (R^2^ = 0.91) at the 60th training epoch. This indicates a significant enhancement in the model's reliability for predicting the potential of herbal pairs (Fig. 3A). Based on the predicted Herbal Combination Potential Index, we further screened pairs with HCPI values greater than 0.5, designating these as “high-potential herbal pairs” (Fig. 3B).

To analyze the characteristics and interrelationships of these high-potential herbal pairs, we constructed a herbal pair network (Fig. 3C), where nodes represent individual herbs and edges indicate potential interactions between herbs. Herbal pairs with higher HCPI values are displayed with thicker or darker lines, allowing for a more intuitive identification of combinations with strong interaction potential. We identified several key nodes (herbs) in the network with frequent pairings with other herbs. These frequently occurring herbal pairs may play significant roles in enhancing therapeutic effects or specific treatment outcomes. We further focused on this network to extract the core high-potential herbal pairs for in-depth analysis (Fig. 3D).

These frequently occurring herbal pairs not only reveal potential synergistic relationships among different herbs but also provide a theoretical foundation for developing new clinical prescriptions. By identifying these pairs with significant interaction potential, we can more effectively design new formulas aimed at optimizing therapeutic efficacy in practical applications.

### Suitability analysis of prescriptions related to colon polyps and intestinal tumors

**Fig. 4:**
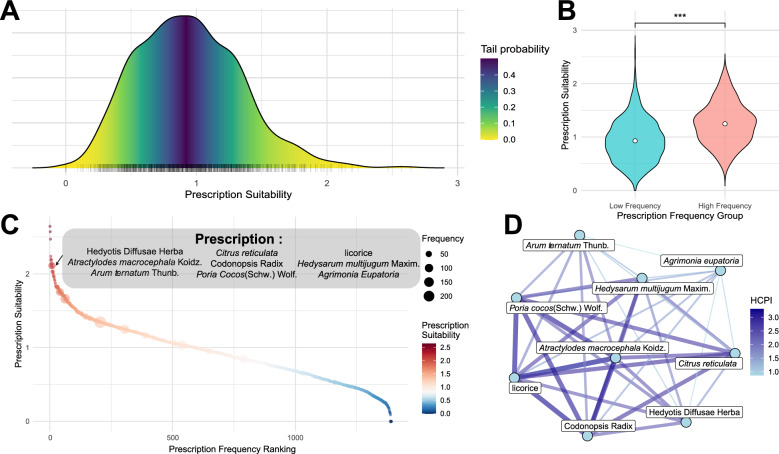
Suitability analysis of prescriptions based on HCPI. **A** Suitability distribution of prescriptions related to colonic polyps and intestinal tuberculosis. **B** Comparison of suitability between high-frequency and low-frequency prescriptions (student t-test, p > 0.05, ns; p < 0.05, *; p < 0.01, **; p < 0.001, ***). **C** Example of a representative high-frequency, high-suitability prescription. **D** Herbal network structure of a representative high-frequency prescription, with nodes representing herbs and edges indicating HCPI values between herbs

We collected and analyzed prescription data from the gastroenterology department of Jiangsu Province Hospital of Chinese Medicine for the years 2022–2023, focusing specifically on prescriptions related to colon polyps and intestinal tumors. After screening, we extracted 3,210 prescription records containing these symptoms from a total of 26,267 treatment records. To quantify the suitability of these prescriptions, we scored each prescription based on previously calculated HCPI values of herbal pairs, yielding a suitability score for each prescription. Most prescriptions showed a suitability concentrated around moderate values, but a few had significantly higher suitability scores than others (Fig. 4A).

In further analysis, we categorized prescriptions into high-frequency (used more than 10 times) and low-frequency groups and compared the suitability distributions of these two groups (Fig. 4B). The results indicate that high-frequency prescriptions have significantly higher suitability than low-frequency ones (p < 0.001), suggesting that prescriptions frequently used in clinical practice have higher suitability and may align better with physicians’ expectations of herbal combination effectiveness.

Additionally, we identified a representative prescription from the data with high suitability and high usage frequency (Fig. 4C). This prescription includes various herbal combinations, such as Hedyotis Diffusae Herb, *Atractylodes* *macroccphala* Koidz., and *Arum* *ternatum* Thunb. The network characteristics of herbs in this prescription exhibit significant interactions, suggesting that these combinations may have synergistic effects that enhance therapeutic outcomes (Fig. 4D).

### Target gene enrichment analysis and protein–protein interaction network study of high-potential prescriptions

**Fig. 5:**
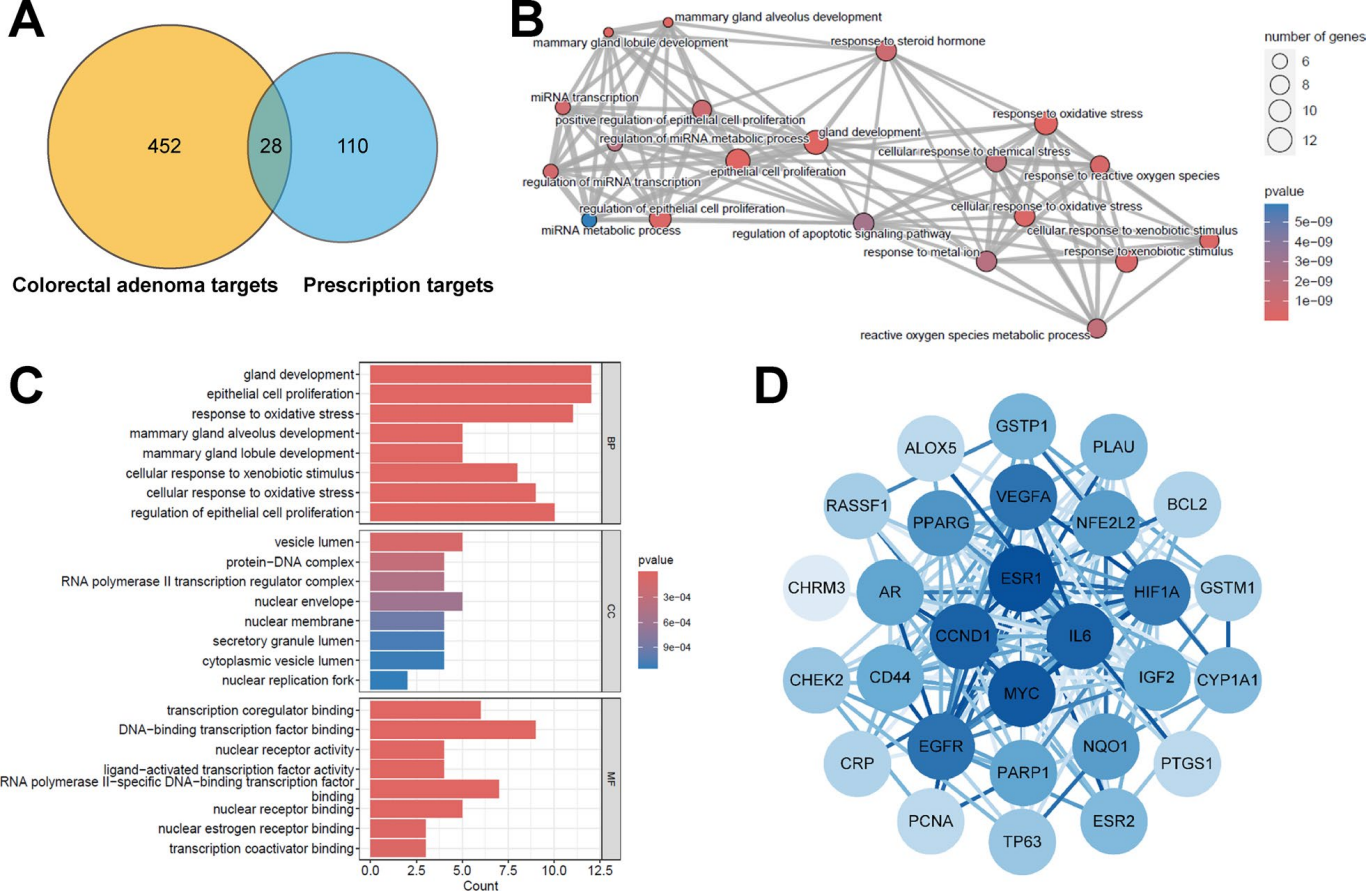
Enrichment analysis of target genes and protein–protein interaction (PPI) network study. **A** Venn diagram of colorectal adenoma targets and high-potential prescription targets. **B**, **C** GO enrichment analysis of overlapping genes. **D** PPI network analysis of overlapping genes

In our further analysis of the potential targets of high-potential prescriptions, we collected known target genes associated with colorectal adenoma and genes targeted by these prescriptions using databases such as TCMSP and Genecard. We identified 28 overlapping genes between the two datasets (Fig. 5A). To better understand the functional roles of these overlapping genes, we conducted a Gene Ontology (GO) enrichment analysis.

The Biological Process (BP) results from the GO analysis revealed that these genes are involved in several critical biological processes, primarily including epithelial cell proliferation, reactive oxygen species (ROS) metabolic processes, cellular response to oxidative stress, response to steroid hormones, and gland development. These processes are closely related to cancer progression and regulation of the cellular microenvironment. Additionally, these overlapping genes are involved in the regulation of miRNA transcription and metabolic processes, suggesting a potential role in the upstream regulation of gene expression (Fig. 5B).

In terms of Cellular Component (CC), these genes are significantly enriched in locations such as the nuclear membrane, protein-DNA complexes, secretory granule lumen, and cytoplasmic vesicle lumen, indicating their potential importance in signaling between the nucleus and cytoplasm and in secretion regulation. At the Molecular Function (MF) level, these genes are significantly involved in transcription factor activity, DNA-binding transcription factor activity, and nuclear receptor activity, highlighting their roles in gene expression regulation and cellular signal transduction (Fig. 5C).

Moreover, we conducted a Protein–Protein Interaction (PPI) network analysis of the 28 overlapping genes. The PPI network revealed a dense interaction pattern, forming a complex network centered on key nodes. Core nodes include IL6, MYC, VEGFA, EGFR, and ESR1, all of which play essential roles in cancer development and may act as critical molecular regulatory hubs (Fig. 5D).

### Screening and validation of active small molecules in high-potential prescriptions

**Fig. 6:**
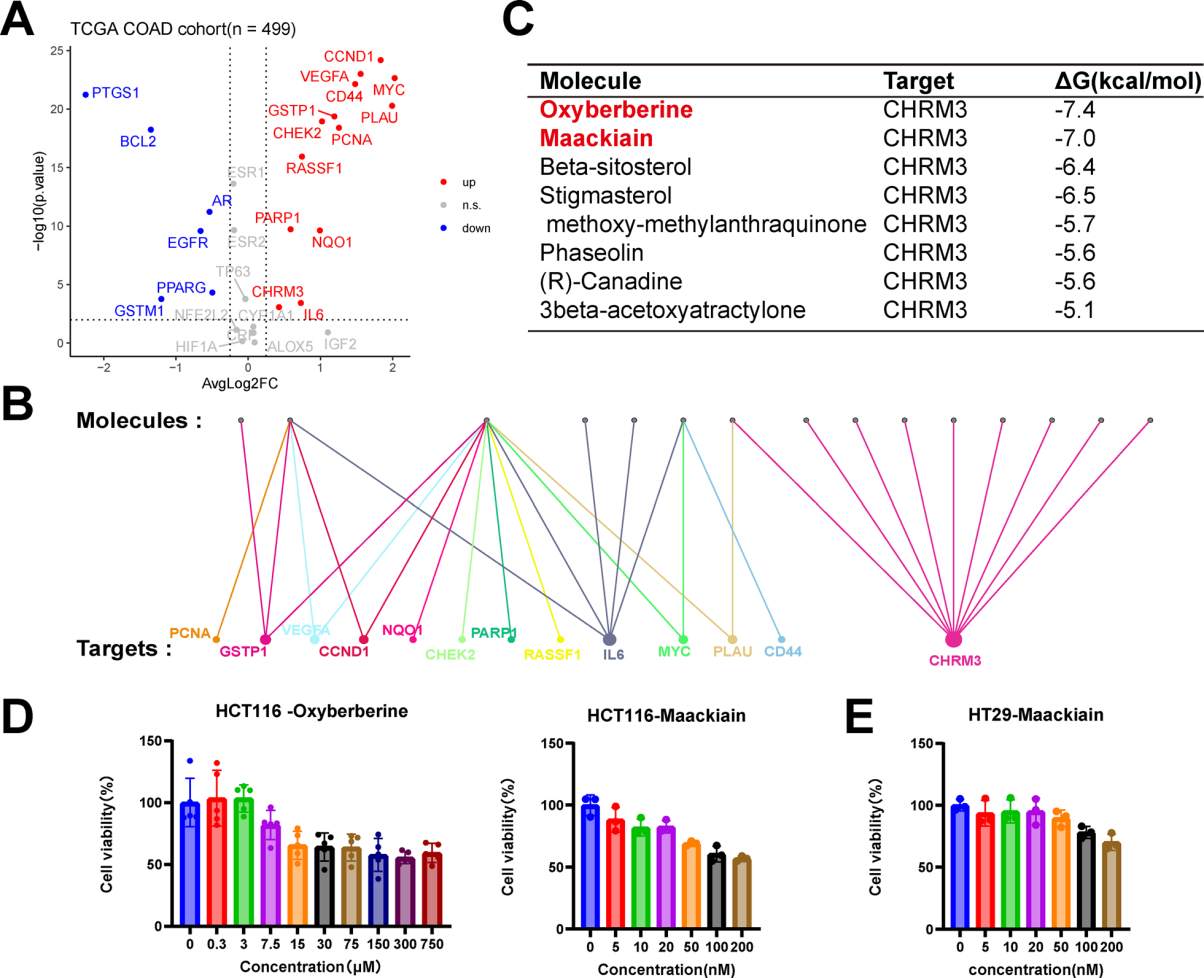
Active small molecules in high-potential prescription. **A** Volcano plot showing differential gene expression. **B** Target frequency analysis in high-potential prescriptions. **C** Molecular docking results of CHRM3. **D**, **E** CCK-8 cell viability assays demonstrating the anticancer potential of small molecules

Based on analysis results from the TCGA database, we further investigated the expression changes of the overlapping genes identified in colorectal cancer. Data showed that 13 overlapping genes were significantly upregulated in colorectal cancer patient tissues (Fig. 6A), suggesting that these genes may play crucial roles in tumor initiation and progression and hold potential as therapeutic targets. Among these 13 genes, CHRM3 was targeted by the highest number of small-molecule compounds across different prescriptions (Fig. 6B), indicating that CHRM3 may be a key molecular target within this prescription.

Through molecular docking experiments, we further analyzed the binding affinities of various small molecules to CHRM3. The results showed that Oxyberberine and Maackiain had the lowest binding energies with CHRM3 (ΔG of − 7.4 kcal/mol and − 7.0 kcal/mol, respectively), indicating that these two small molecules may possess strong binding capabilities (Fig. 6C), which could make them promising candidates for the treatment of colorectal adenoma.

To validate the antitumor activities of Oxyberberine and Maackiain, we conducted CCK8 proliferation assays on the colorectal cancer cell line HCT116. The experimental results indicated a significant reduction in HCT116 cell viability as the concentrations of Oxyberberine and Maackiain increased (Fig. 6D), suggesting that these small molecules have inhibitory effects on colorectal cancer cell proliferation. Further experiments focused on Maackiain, which showed a more pronounced inhibitory effect on the HCT116 cell line. We validated this effect in another colorectal cancer cell line, HT29. The results similarly demonstrated that Maackiain significantly inhibited the viability and proliferation of HT29 cells (Fig. 6E), further supporting its potential as a therapeutic agent for colorectal cancer.

## Discussion:

This study employs a heterogeneous GNN model to systematically analyze the compatibility patterns of TCM prescriptions in the treatment of colorectal adenoma. We propose the HCPI as a quantitative measure of herbal compatibility and further develop a prescription efficacy prediction method. This model demonstrates significant advantages in elucidating herbal interactions and compatibility, providing a scientific foundation for optimizing TCM-based treatment strategies for colorectal adenoma.

From clinical records, we have identified a high-potential prescription comprising Hedyotis Diffusa Herba, *Atractylodes macrocephala* Koidz., and *Arum ternatum* Thunb. Network pharmacology analysis suggests that Maackiain may serve as a key active compound. Preclinical studies have already demonstrated the therapeutic potential of Maackiain, and existing literature has reported its efficacy in the treatment of endometrial cancer [21, 22]. Moreover, Maackiain has shown promising clinical applications in the management of benign prostatic hyperplasia [23]. Based on these preliminary findings, future research will focus on further elucidating the mechanistic role of Maackiain in colorectal adenoma treatment and facilitating its translation into clinical applications.

Despite these initial advancements, the model still faces several challenges. Its predictive accuracy is contingent on the quality and completeness of prescription and ingredient data. Given the complexity of colorectal adenoma pathology and its diverse clinical manifestations, the current dataset may not fully encompass all relevant cases. To enhance the robustness of the model, expanding the dataset to include a broader range of clinical records and patient populations will be necessary to improve generalizability.

Additionally, individual variability and the dynamic progression of disease pose challenges for a static predictive model. TCM prescriptions are often adjusted based on patient-specific conditions, a factor that is difficult to capture in a fixed model. Future optimizations should incorporate personalized variables such as genetic profiles, age, and comorbidities, allowing for more precise and individualized treatment recommendations [24, 25].

Furthermore, while the model effectively analyzes herbal interactions, it remains limited in its ability to fully characterize the synergistic and antagonistic effects within herbal prescriptions. Integrating molecular pharmacology data to elucidate the impact of active compounds on specific signaling pathways could enhance the scientific rigor of prescription recommendations, ensuring a balanced consideration of both efficacy and safety.

Future research should also extend the model’s applicability to other diseases, exploring optimization strategies for TCM prescriptions in various clinical contexts. Integrating multi-omics data, including genomics and transcriptomics, could further enhance predictive precision, while dynamic learning mechanisms would enable continuous model refinement as new clinical data become available. With ongoing improvements and validation, this approach holds great potential for advancing personalized TCM treatments, not only in colorectal adenoma but also in broader disease areas.

## Conclusion

In summary, this study uses an GNN model to systematically predict compatibility patterns in TCM prescriptions and employs network pharmacology to preliminarily elucidate the mechanisms of active ingredients in TCM combinations. This research not only provides theoretical support for TCM treatment of colorectal adenoma but also offers technical guidance for future TCM formulation design and efficacy optimization.

## Data Availability

The datasets used and/or analyzed among the current study are available from the corresponding author on reasonable request.
